# Content Validity and Psychometric Properties of the Nomination Scale for Identifying Football Talent (NSIFT): Application to Coaches, Parents and Players

**DOI:** 10.3390/sports5010002

**Published:** 2017-01-01

**Authors:** Alejandro Prieto-Ayuso, Juan Carlos Pastor-Vicedo, Onofre Contreras-Jordán

**Affiliations:** Research Group EDAF, University of Castilla-La Mancha, Albacete 02071, Spain; JuanCarlos.Pastor@uclm.es (J.C.P.-V.); Onofre.CJordan@uclm.es (O.C.-J.)

**Keywords:** football, talent, scale, scouting, instrument

## Abstract

The identification of football talent is a critical issue both for clubs and the families of players. However, despite its importance in a sporting, economic and social sense, there appears to be a lack of instruments that can reliably measure talent performance. The aim of this study was to design and validate the Nomination Scale for Identifying Football Talent (NSIFT), with the aim of optimising the processes for identifying said talent. The scale was first validated through expert judgment, and then statistically, by means of an exploratory factor analysis (EFA), confirmatory factor analysis (CFA), internal reliability and convergent validity. The results reveal the presence of three factors in the scale’s factor matrix, with these results being confirmed by the CFA. The scale revealed suitable internal reliability and homogeneity indices. Convergent validity showed that it is teammates who are best able to identify football talent, followed by coaches and parents. It can be concluded that the NSIFT is suitable for use in the football world. Future studies should seek to confirm these results in different contexts by means of further CFAs.

## 1. Introduction

Talent identification is vital to the unearthing of future sports stars [1,2], and football is no exception to that rule. In the football world, sporting and economic objectives essentially dictate the way in which the processes for selecting football players from young ages [3] are conducted. An example of the sporting objective is provided by F.C. Barcelona (named the best training club in a 2015 report by the CIES Football Observatory), which admits only 0.5% of the 10,000 children it watches every year to its La Masía academy [4]. However, despite the small number of children who meet the club’s selection criteria, Guillermo Amor, one of the heads of its youth academy, says that “our intuition fails many us, and a lot” [4] (p. 27). Meanwhile, at Real Madrid (which received an award from *France Football* in 2008 for having the best youth setup in Europe) economic objectives are a cornerstone of its youth training policy [5], with the club seeing “business opportunities in young football players” [5] (p. 38).

Talent identification has thus become a key issue in the sport, for both the majority of the world’s clubs, in their desire to achieve their objectives, and for the families of players, given the rise in social status that footballers enjoy on reaching the elite [6]. Regardless of the talent management model implemented by each club [7], it goes without saying that the instruments used in processes for identifying football talent should be reliable, due to the considerable importance they have in sporting, economic and social terms.

Nonetheless, the various existing tests used to measure talent performance [8] do not appear to be clearly defined [9], mainly due to the lack of objective criteria for gauging player performance [10]. The difficulty with the identification process lies in that fact that the successful selection of players requires looking beyond the point at which they are evaluated [4]. The skill lies in identifying players who do not attract attention, but who have the potential to reach the elite. These players are the most difficult to spot. This is the main reason why football coaches pose a problem when it comes to setting criteria for the identification of the most talented players [11], criteria that are based mainly on intuition [4].

There have traditionally been two approaches to measuring talent performance [12]. The first of them, the top-down approach, seeks to analyse, from a quantitative viewpoint, the characteristics of elite athletes, with the aim of assessing and predicting performance. The work carried out by Matthys et al. [13], Torres-Unda et al. [14] or Zhanel et al. [15] are examples of such an approach, where the anthropometric, physical, physiological, technical, tactical and psychological characteristics of athletes are taken as reference points for assessing and predicting performance. There are football-specific studies that have sought to identify the differences between expert and novice players at an anthropometric [16], physical, physiological [17] and cognitive [18,19] level. We have also come across studies that seek to identify the best performance predictors [20] based on the functional capabilities of the footballer [21,22] and their physiological characteristics [23,24]. There are also training programmes that aim to respond to the individual needs of each player by working on skills such as physical speed, agility and speed of thought [25]. These programmes are conducted with professional football players [26] or as part of the personalised training sessions organised by the Fédération Internationale de Football Association (FIFA) for talented players [27]. Finally, this approach obtains indicators of the performance of such players, both individually [28] and collectively [29].

Nevertheless, despite the potential advantages provided by this quantitative approach, it does involve two limitations. One stems from the type of study population, which is usually expert/novice, inherent in which is the subjective nature of the label “expert”. The other is the type of design. There is a lack of longitudinal studies [21], which can mean that the results obtained at any given moment are conditioned by factors such as maturity or the relative age effect [30].

The aim of bottom-up, the second of the two approaches to measuring talent performance, is to study the most qualitative aspects determining the training and development of football talent [19]. This approach is a response to the difficulty of objectifying the identification process and the limitations posed by the quantitative approach [21]. The importance of the qualitative study of performance lies in the individual knowledge possessed by each player, which, combined with practice, creates a unique path in the talent development process [31]. The studies conducted by Bloom [32] and Coté [33] are examples of such work and involved interviews with athletes reaching the elite and with their families. Also notable in this respect is the work carried out by Fiorese Vieira, Lopes Vieira and Jornada Krebs [34], who used a case study to chart the career path of an Olympic swimming champion; a study conducted by Sánchez Sánchez [35], who interviewed professional basketball players; and the work of Cabanillas Cruz [36], who reconstructed the life story of a professional karateka from a case study. Similar studies have been conducted in football, such as one undertaken by Matthew and Harwood [37], who analysed the performance context of young English footballers by carrying out interviews with coaches, experts and players. In a similar vein, Pazo Haro, Sáenz-López Buñuel, Fradua Uriondo, Barata Figueiredo and Coelho e Silva [38] used interviews to study the development of football players from the perspective of youth academy coordinators. These studies reveal the existence of myriad contextual factors, such as the family, practice opportunities, injuries and even luck, all of which can be crucial to achieving sporting success. The limitations of this approach arise mainly from the context in which research is carried out, due to the range of sports played, which varies according to the country of birth [39], and the lack of transnational studies that would otherwise compensate for this limitation.

To sum up, what is striking in the football world is that while the motives for the correct identification of talent appear to be clear (mainly sporting and economic) [3], there does not appear to be a clearly defined instrument enabling a quality talent identification process to be carried out [9]. Furthermore, even in the physical tests used to identify talent, it has been shown that there is no correlation between their results and the final selection of athletes [40]. One study confirming this finding was carried out by Lago-Peñas, Rey, Casáis and Gómez-López [20]. It established the physical (speed, strength and stamina) and anthropometric profiles (height, weight, body mass index, six skin-folds, four diameters and three circumferences) of young football players in accordance with their position (16 goalkeepers, 26 central defenders, 29 full-backs, 34 defensive midfielders, 28 midfielders and 23 forwards) through physical tests such as the Yo-Yo Test, Sprint Test and Jump Test. At the end of the season, the coaching staff was asked to select which players should stay on in the team, with the rest being excluded. The results of the study confirm that body composition was similar in the two groups. Though the selected players had slightly better physical attributes, the statistical differences between them and the excluded players were not significant.

Educational sources are the only ones that provide any successful precedents regarding the use of objective instruments (nomination scales or intelligence tests) for measuring talent performance [41], with it being noted that the triangulation of information from teachers, parents and teammates provides a very reliable means for identifying giftedness [42,43]. It seems obvious to think that the teacher is an adequate agent to perform this type of detection, however, it has been demonstrated for decades that parents are good and effective identifiers of students with high intellectual abilities [44], having been showed his reliability for these processes [45]. Therefore, due to the findings found in these studies, current studies [46,47,48,49] include the figures of the expert (teacher), parents and classmates in their processes of detection of students with high intellectual abilities, because these may contribute by informing the school of their child's abilities that cannot be detected by the teacher at school. This, together with the possible biases committed by the teachers [50], give the rest of the parents and peers a great importance in this process.

Subsequently, just as is the case in the educational environment, where scales and intelligence tests are focused on measuring a specific aspect pertaining to a gifted student (a talent for mathematics, speaking, music etc.), in the football world it is the areas that are most crucial to becoming a talented footballer that must be identified first of all. In accordance with the rules and their internal logic, therefore, and as shown in the literature consulted, the most decisive aspects in identifying a talented footballer are technical/tactical aspects such as decision-making and positioning [51,52], behavioural aspects such as discipline [53], mental aspects such as game intelligence and attitude [11], and social attributes such as the ability to engage with teammates [38].

As a result, and in view of the above, the aim of this study is to design and validate an instrument that can be used to assess football players and that brings about an improvement in the football talent identification and development processes so that optimal use may be made of the resources football clubs allocate to this task.

## 2. Method

### 2.1. Sample and Context

The total sample was 366 subjects, comprised of football coaches (3%), parents of the football players (42.07%) and the footballers themselves (54.91%). The football coaches completed the scale in relation to all the players in their teams, the parents of the players completed the scale in relation to their sons, and the football players completed the scale in relation to their teammates. A total of 556 questionnaires were collected from the three aforementioned groups: coaches, parents and players.

The study was conducted with youth teams at a professional club, with 30 of the players being under 10s (U10s), 29 U12s, 38 U14s, 37 U16s, 47 U19s and 20 of them being U23 amateur players. The average age was 13.67 (SD = 2.97).

The study was approved by the Clinical Research Ethics Committee (CEIC) of the University of Castilla-La Mancha, with this approval being recorded in Report No. 03/2016 of the CEIC. The club and the parents also gave written informed consent allowing the players to take part in the study, while the researchers guaranteed that the data they collected would be treated confidentially, in accordance with Organic Data Protection Act 15/1999 [54], dated 13 December.

### 2.2. Instrument

In creating the scale, a decision had to be made, first of all, as to the dimensions and items to be included, or as to which aspects were relevant to the identification of football talent. A review of the literature on the development of football talent revealed the most important dimensions. In line with direct education-related precedents, it was decided to use a Likert-type response scale, as this was deemed to be the most suitable means of carrying out the measurements. The researchers then engaged in a validation process with 16 experts, whose selection criteria were in the case of researchers, be licensed in Physical Activity and Sport Sciences, doctor and with extensive experience (more than ten years) in the training of football players. And in the case of coaches, have more than ten years experience in professional football clubs. In total, there were eight researchers and eight football coaches who met the requirements described. The questionnaire was sent to the experts in a table of specifications so that the percentage awarded to each dimension could be assessed, along with the relevance of each of the items proposed. Finally, an exploratory and confirmatory factor analysis was carried out in order to ascertain the structure of the factor matrix.

The result was a scale comprising three dimensions: (1) cognitive aspects, relating to game intelligence and problem solving; (2) psychological aspects, relating to sport commitment and the ability to shoulder responsibilities; and (3) motivation, relating to the desire to improve as a player.

### 2.3. Procedure

Data was collated by means of a scale for identifying football talent in youth categories with the aim of ascertaining which players were nominated by their teammates, parents and coaches. The scale was handed out to the coaches and parents, who completed it on their own, while the players completed it individually, with the assistance of a researcher. Parents and coaches were asked to indicate the extent to which they agreed or disagreed with each of the items. A five-point Likert scale was used: 1—strongly disagree; 2—disagree; 3—neither agree nor disagree; 4—agree; 5—strongly agree. The players were asked to name which teammate stood out most in each of the items.

The procedure involved randomly dividing the sample into two subsamples: (1) a calibration sample (*n* = 277) and (2) a validation sample (*n* = 279). The first sample was used to select the items (in conjunction with content validity) and the second to assess the psychometric properties of the scale. The factor analysis requires that the sample is equal to or larger than 150 cases and that there is a correlation between the variables under analysis [55].

### 2.4. Data Analysis

Expert judgment was used to analyse the content of the scale, in line with the validity criteria proposed by Lashwe [56]. A total of 16 experts took part, eight from each of the two following groups: (a) researchers (doctors) in the field of physical activity and sports sciences; and (b) Level 1/2/3 football coaches.

**Figure 1:**
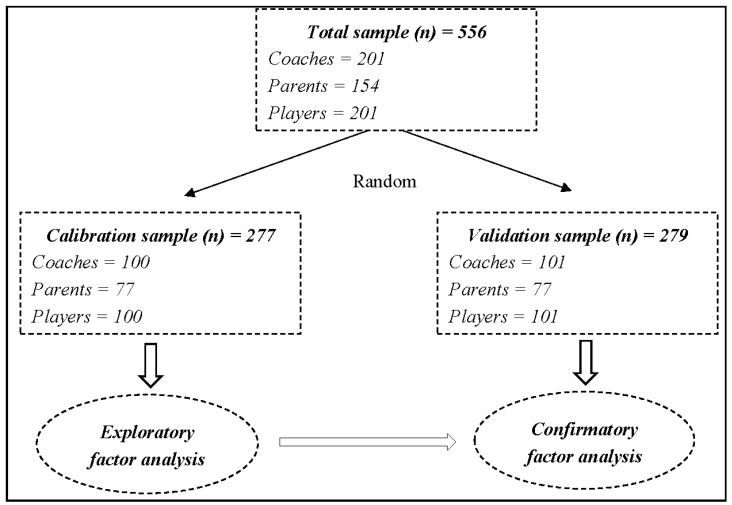
Research design of the study.

Statistical analysis of the data involved, first of all, an exploratory factor analysis (EFA) for ascertaining the factor structure of the scale. An internal consistency analysis of the scale and of each of the factors was also conducted to determine the reliability coefficient with Cronbach’s alpha [57]. Finally, a confirmatory factor analysis (CFA) was conducted with structural equations to check how the data fitted the theoretical model. SPSS v. 22.0 and Amos 21.0 software were used for the EFA and CFA respectively, using a 95% confidence interval. Figure 1 represents the research design of the study.

## 3. Results

Table 1 shows the Content Validity Index (CVI) as proposed by Lashwe [56] for each of the items on the original scale.

In accordance with Laswhe’s [56] criteria, items 2, 10, 11, 16, 21, 22, 23, 24, 25, 26 and 27 were removed. Despite showing a lower value, items 1 and 13 were retained in the final scale because of their importance to the study and the wealth of literature on sport commitment [30,31,56] as a decisive factor in football talent. Table 2 shows the final scale. It includes a brief explanation of each item, another recommendation made by the experts.

Secondly, an exploratory factor analysis of principal components with Varimax rotation (Table 3) was carried out with the resulting 13 items in the calibration sample. As there is no suitable theory about the aspect under analysis, the aim being, therefore, to check the factor matrix of the scale.

To check that the data matrix is suitable for conducting an EFA [51], Bartlett and Kaiser, Meyer and Olkin’s test (KMO) of sphericity was carried out, with appropriate values being obtained in the replies of the coaches (0.871), parents (0.825) and players (0.777).

An EFA was then conducted to check the number of factors in the scale, with the answers being separated into different categories: coaches, parents and players. Table 3 shows how the items are distributed in each factor in accordance with the coaches’ answers.

Table 4 shows the factor loads in accordance with the answers given by the parents of the players.

Table 5 shows the factor loads in accordance with the answers given by the players themselves.

The factor analysis pointed to the existence of three factors that explain the variance of the matrix. The first factor corresponds to game intelligence (technical/tactical aspects). The second factor relates to motivational aspects. The third factor corresponds to psychological aspects.

The internal consistency of the scale was calculated using Cronbach’s alpha coefficient. The questionnaire delivered to the coaches revealed a total coefficient of 0.906 for Factor 1, 0.911 for Factor 2, and 0.848 for Factor 3. The internal consistency of the general scale for coaches was 0.914.

In the case of the players’ parents, the internal consistency of the scale revealed values of 0.838 for the first factor, 0.758 for the second factor, and 0.583 for the third factor. The internal consistency of the general scale for the players’ parents was 0.869.

Finally, the internal consistency of the scale in accordance with the answers given by the players revealed values of 0.784 for Factor 1, 0.745 for Factor 2, and 0.659 for Factor 3. The internal consistency of the general scale for the players was 0.852.

Thirdly, a confirmatory factor analysis was conducted with the validation sample, using Amos 21.0 software. The maximum likelihood method was used to estimate the parameters. The following fit indices were used: X^2^, the Standardized Root Mean Squared Residual (SRMR), the Root Mean Square Error of Approximation (RMSEA), the Tucker-Lewis Index (TLI), and the Comparative Fit Index (CFI). The results are shown in Table 6.

These values indicate that, in general, the model fits the data, thus confirming the theoretical structure proposed.

Fourthly, for the purposes of assessing the convergent validity of the NSIFT, a correctional analysis was made of the scores awarded by the three groups studied (coaches, parents and players) and the relation with the final result (nomination as a talented player). The results are shown in Table 7.

Table 7 shows a significant (*p* < 0.001), moderate (between 0.41 and 0.6) and positive correlation between the results obtained for the coaches and the players (0.434). Furthermore, the three groups correlate significantly with the nomination of football talent, with the relation with parents being moderate (0.499), the relation with coaches high (between 0.61 and 0.8, in this particular case 0.711) and the relation with players very high (between 0.81 and 1, in this particular case 0.847).

Finally, Table 8 shows the correlations between the three factors of the matrix.

Table 8 shows how, in the case of coaches and players, all the relations between factors are positive and moderate (between 0.41 and 0.6). With regard to the players, however, the relation between their factors is slightly lower. Regarding the parents, meanwhile, the relations are negative and their intensity is low and moderate.

## 4. Discussion

The bibliographical search for precedents with regard to the identification of football talent underlines the argument put forward by [9], who hold that it is unclear which tests are suitable for satisfactorily measuring talent performance in football. With that in mind, the aim of this study was to design and validate an evaluation instrument (NSIFT) for identifying football talent, the objective being to improve the processes whereby footballers are identified so that full use can be made of the resources allocated by clubs to this task, which is one of great importance [6].

In designing the NSIFT, the study’s researchers drew on the prior literature consulted, and identified the technical/tactical dimension [51,52], character [53], mental approach [11] and social attributes [38] as the most decisive aspects with regard to football players. The scale was then validated by the judgment of 16 experts (eight doctors from the field of physical activity and sports sciences and eight football coaches) with a view to determining if these items fit in with the reality measured, that of football talent. Lashwe’s [56] criteria were followed in obtaining the CVI of each item from the answers of each expert individually in a table of specifications.

The results obtained from the answers given by the experts in validating the NSIFT confirm the importance attached in the prior literature to cognitive [51,52], psychological and social [38,53,58] and mental dimensions [59] in identifying a talented prospect in this field, which supports the findings of Miller, Cronin and Baker [60], as it is not entirely clear that the physical dimension is an accurate indicator of football talent [20].

Secondly, exploratory and confirmatory analyses were conducted with a calibration and validation sample respectively, chosen at random from the sample used for this study. The aim was to determine the structure of the factor matrix of the scale and thereby ascertain the factors into which the instrument was divided, and to check if these results coincided with the prior literature consulted and the expert judgment. The results of the analysis confirmed that the factor structure of the scale is comprised of three factors, which tie in with those proposed by the experts: game intelligence (cognitive aspects), motivational aspects and psychological aspects. It should be pointed out that the lack of prior scales of this type in football means that a comparison could not be made. Furthermore, and continuing with the statistical analysis of the instrument, an internal reliability analysis was carried out (Cronbach’s alpha) with the aim of determining its reliability. The results of the analysis indicated high reliability values (all above 0.8) for the three aforementioned groups (coaches, parents and players). The literature indicates that all instruments with a value lower than 0.7 can be seen as being largely unreliable [57]. The results obtained in this study therefore reveal a high consistency between the variables in the scale. This confirms the importance of including coaches, parents and players in football talent identification processes, as stated in the only precedents from the educational environment [41,42,43,44,45,46,47,48,49,50]. If this were not the case, these processes would be based solely on the intuition of the expert [4]. The use of nothing but expert opinion would result in a large number of footballers with potential talent being left out of the “talent pool”, as Renzulli and Gaesser [44] call it.

The convergent validity of the NSIFT revealed statistically significant relations between the nomination made by the players and the coaches. This is not the case with the parents. This can be explained by the fact that it is the coaches and team members who possess greater knowledge of the players, as they spend more time with them in the football environment. Another of the study’s findings confirming this is the fact that the players are best able to identify talented teammates, achieving a higher correlation (0.847) than that obtained by coaches and parents, which indicates that the people who spend the most time together in this environment are able to more clearly identify who stands out in each facet of the game. This finding confirms the precedents found in the educational environment, in which in most of the instruments designed for the nomination of gifted students—though it is true that they consider the triangulation of information from teachers, classmates and parents [45,46,47,48,49,50]—the first step in the screening process involves ascertaining the opinion of teaching staff. This is the case, for example, in Renzulli’s Identification System for Gifted Programming Services (RIS/GPS) [42], in which teacher and test-based nomination are the first two steps in creating the talent pool, due to their importance.

Finally, a correlational analysis of the scale was conducted with the aim of finding out the relation between each of the three factors identified in the factor matrix, according to the answers given by the coaches, parents and players. The results showed that the relation between the factors of the players and coaches was similar. However, with regard to the parents, the relation between Factor 1 (game intelligence) and Factor 2 (mental aspects) is negative, which indicates that as the cognitive abilities (game intelligence) of a football player increase, their motivation to play football decreases. This can be explained by the change in the role of families in the development of talented players, given that, and in accordance with the Developmental Model of Sport Participation (DMSP) proposed by Coté [33], as the athletes' sport commitment increases, so the coach takes on an increasingly prominent role, with the role of the family decreasing. In light of these findings, and bearing in mind that it is parents who are least capable of identifying football talent, we can conclude that the rather remote vision they possess provides an explanation as to why they think that as a player progresses in their age group, their motivation with regard to the sports environment can fall away, this aspect being vital for sport performance [61].

In short, this study confirms the reliability of the scale proposed for the processes of identifying and developing football talent, and emphasises the importance of including both parents and players in the process, with it being shown that teammates are best equipped to identify football talent, followed by coaches (experts) and parents. As a result, these identification processes cannot be based almost exclusively on the intuition of the expert [4], given that their intuition can “fail” many of them and on many occasions, as Amor states [4] (p. 27). Furthermore, these results confirm those of the sources from the educational environment, in which the triangulation of information has been used for many decades to identify gifted students. An example of this is the study conducted by Sánchez López [62], the objective of which was to identify gifted students at schools through the opinion (scales) of teachers, parents and classmates. Another is Renzulli and Gaesser’s aforementioned model [44].

## 5. Conclusions

As we have seen in the existing literature, the football world needs to improve talent identification instruments so that selection processes can be perfected and refined. This is the objective pursued by the study, and from it we can draw conclusions that we believe to be of some importance, as they help address its stated goal. We have found that the most important dimensions in the identification of football talent are cognitive (technical/tactical aspects), mental and psychological in nature. It is for this reason that the processes for identifying young players and their development through the various age groups should be focused not only on improving technical/tactical performance but also on mental aspects such as continued motivation towards football, and psychological aspects such as the development of social ties with the rest of the player’s teammates, which are crucial to achieving success. We have also noted how teammates are best able to identify talented players, followed by experts (coaches) and parents, which highlights the importance of direct contact on a daily basis. The development of the player though the various age categories should not, therefore, be founded exclusively on their biological age or the clinical eye of the coach, but also on the opinion of their teammates, which can be crucial in convincing the club that the player in question has a chance of continuing their journey to the elite. Thirdly, the scale is shown to be a reliable instrument for use with coaches, parents and players. It can thus be concluded that this instrument will help clubs to assess football talent through the triangulation of information between experts (coaches), the parents of players and the players themselves, with the purpose of optimising the resources allocated by clubs to this task.

## 6. Limitations

Rigorous monitoring of the scientific method in carrying out this study revealed certain limitations deriving from the way in which the sample was surveyed. In some cases this was done by email, as some of the teams in the study had finished their regular seasons, which meant it was not possible to conduct the survey face-to-face. Another limitation we encountered was that some parents did not want to take part in the study. In some cases this was because they did not feel capable of filling in the scale due to their lack of football knowledge, and in others because they did not want to provide details about their children (date of birth, height, weight). As a result, their data was removed from the study. Another limitation was the experimental mortality of subjects, which in this case was related to the incidence of injuries sustained as a result of playing the sport that is the subject of the study. Subsequently, the size of some of the group samples was reduced, as the players who suffered injuries during the period covered by the study could not take part in it.

## 7. Prospects for Research

There are two avenues for future research. Firstly, there is the need to implement the scale in other contexts, the aim being to ascertain if the factor matrix resulting from this study (cognitive factor, mental factor and psychological factor) can be confirmed. Secondly, there is the need to reuse the scale in the same context as this research study, with the aim of finding out if it can achieve its objective—the identification of talent—in a longitudinal manner.

## Figures and Tables

**Table 1:** Content Validity Index (CVI) for each item on the original scale.

ITEM		CVI
Cognitive dimension		
1	Interprets the coach’s instructions correctly	0.46
2	Moves into space to support teammates	0.33
3	Anticipates the play on occasion	0.86
4	Takes the correct decision in each situation	0.86
5	Thinks creatively	0.46
6	Executes skills quickly	0.73
7	Able to read the game clearly and quickly	0.86
8	Possesses excellent positional sense (creates and uses space)	0.60
9	Possesses good overall vision of where their teammates and opponents are	0.60
10	Able to link up with teammates	0.20
11	Takes responsibility by performing tasks	0.20
Psychological dimension		
12	Disciplined	−0.06
13	Makes an effort in matches and training	0.46
14	Motivated when it comes to matches and training	0.46
15	Keen to learn and develop	0.73
16	Able to lead their teammates	−0.20
17	Able to concentrate in matches and training	0.86
18	Possesses a winning mentality	0.86
19	Has a positive attitude	0.60
20	Willing to take on responsibilities	0.73
Social dimension		
21	Displays a sense of belonging to the team: plays for the rest of the team	0.20
22	Plays the game sportingly (fair play)	−0.46
23	A good communicator (shows respect for others, is sociable, cooperates, a good listener)	0.06
24	Knows the difference between having fun and training	0.20
25	Approachable with their teammates	−0.33
26	Popular with their teammates and has their support	0.20
27	Finds it easy to get on with others	−0.33

**Table 2:** Final scale.

**1**	**Interprets the coach’s instructions correctly**
	Relates to the player’s ability to understand and correctly implement the instructions given by the coach (set-piece routines, strategy, style of play, etc.).
**2**	**Usually anticipates play**
	Relates to the player’s ability to read the opponent's intentions, position themselves to receive the ball and stay alert during games.
**3**	**Generally makes the right decision**
	Relates to the player’s ability to make the correct choices during games, both when they are on the ball (passing, dribbling etc.) and off it (covering, moving into space etc.).
**4**	**Executes skills very quickly**
	Relates to the player’s ability to execute a technical skill quickly and correctly in resolving to a tactical situation.
**5**	**Able to read the game clearly and quickly**
	Relates to the player’s ability to know where teammates and opposing players are when they are on the ball so that they can make the best possible decisions in the shortest possible timeframe.
**6**	**Has good positional sense**
	Relates to the player’s ability to take up a suitable position when not in possession of the ball and thereby give balance to their team. This can be both in attack (creating space) and in defence (providing cover or dropping back).
**7**	**Knows where their teammates are on the pitch**
	Relates to the player’s ability, both when on the ball and off it, to know where their teammates are situated. This can be both in attack (the ability to start a counter-attack) and in defence (taking up the correct position).
**8**	**Makes an effort in matches and training**
	Relates to the player’s ability to exert themselves (both in terms of motivation and responsibility) in training sessions and in matches.
**9**	**Keen to learn and develop**
	Relates to the player’s ability to push themselves to be a better player and to take the coach's advice on board.
**10**	**Able to concentrate in matches and/or training**
	Relates to the player’s ability to concentrate both during matches and training sessions (ability to cope with pressure in both scenarios).
**11**	**Possesses a winning mentality**
	Relates to the player’s intrinsic desire to be a winner, both in training and in matches.
**12**	**Has a positive attitude**
	Relates to the player’s ability to be positive in thought and to urge teammates on when necessary and help them to improve.
**13**	**Willing to take on responsibilities**
	Relates to the player’s ability to take on the responsibilities that come with the age group, such as accepting the captaincy or taking a penalty. Responsibilities vary depending on the age group, as the objective at younger ages is to play and have fun.

**Table 3:** Factor loads of the matrix rotated (coaches) using the Varimax method.

Item	Factor 1. Game Intelligence	Factor 2. Motivation	Factor 3. Psychological
1. Interprets the coach’s instructions correctly	0.742		
2. Usually anticipates play	0.710		
3. Generally makes the right decision	0.790		
4. Executes skills very quickly	0.617		
5. Able to read the game clearly and quickly	0.823		
6. Has good positional sense	0.611		
7. Knows where their teammates are on the pitch	0.754		
8. Makes an effort in matches and training		0.852	
9. Keen to learn and develop		0.883	
10. Able to concentrate in matches and training		0.794	
11. Possesses a winning mentality			0.645
12. Has a positive attitude			0.581
13. Willing to take on responsibilities			0.968

**Table 4:** Factor loads of the matrix rotated (parents) using the Varimax method.

Item	Factor 1. Game Intelligence	Factor 2. Motivation	Factor 3. Psychological
1. Interprets the coach’s instructions correctly	0.457		
2. Usually anticipates play	0.455		
3. Generally makes the right decision	0.654		
4. Executes skills very quickly	0.710		
5. Able to read the game clearly and quickly	0.810		
6. Has good positional sense	0.565		
7. Knows where their teammates are on the pitch	0.660		
8. Makes an effort in matches and training			0.785
9. Keen to learn and develop			0.779
10. Able to concentrate in matches and training		0.619	
11. Possesses a winning mentality		0.611	
12. Has a positive attitude		0.859	
13. Willing to take on responsibilities		0.741	

**Table 5:** Factor loads of the matrix rotated (football players) using the Varimax method.

Item	Factor 1. Game Intelligence	Factor 2. Motivation	Factor 3. Psychological
1. Interprets the coach’s instructions correctly	0.449		
2. Usually anticipates play			0.633
3. Generally makes the right decision	0.722		
4. Executes skills very quickly			0.706
5. Able to read the game clearly and quickly	0.795		
6. Has good positional sense	0.696		
7. Knows where their teammates are on the pitch	0.786		
8. Makes an effort in matches and training			0.645
9. Keen to learn and develop		0.791	
10. Able to concentrate in matches and training		0.706	
11. Possesses a winning mentality			0.627
12. Has a positive attitude	0.318		
13. Willing to take on responsibilities		0.780	

**Table 6:** Goodness-of-fit indices.

	Coaches	Parents	Players
X^2^	157.570 (*p* < 0.001)	86.781 (*p* < 0.05)	166.746 (*p* < 0.001)
CFI	0.89	0.89	0.79
TLI	0.87	0.84	0.73
RMSEA	0.12	0.07	0.12

**Table 7:** Correlations matrix (Spearman’s rho).

	Parents	Coaches	Players
Parents		0.148	0.139
Coaches	0.148		0.434 **
Players	0.139	0.434 **	
Talent Nomination	0.499 **	0.711 **	0.847 **

** *p* < 0.001.

**Table 8:** Correlations between the three factors of the matrix.

Factor	Coaches			Parents			Players		
	1	2	3	1	2	3	1	2	3
1		0.450	0.510		−0.535	−0.332		0.342	0.350
2	0.450		0.485	−0.535		0.292	0.342		0.132
3	0.510	0.485		−0.332	0.292		0.132	0.132	
